# Glass Ceramic Fibers Containing PbS Quantum Dots for Fluorescent Temperature Sensing

**DOI:** 10.3390/nano14100882

**Published:** 2024-05-19

**Authors:** Tingyu Zha, Penghui Zhang, Xilong Jin, Yi Long, Taoyun Huang, Hong Jia, Zaijin Fang, Bai-Ou Guan

**Affiliations:** 1 Guangdong Provincial Key Laboratory of Optical Fiber Sensing and Communications, Institute of Photonics Technology, Jinan University, Guangzhou 510632, China; zz2419917512@stu2021.jnu.edu.cn (T.Z.); phzhang@stu2022.jnu.edu.cn (P.Z.); xilongjin2017@jnu.edu.cn (X.J.); longyi9604@stu2019.jnu.edu.cn (Y.L.); taovinhuang@stu.jnu.edu.cn (T.H.); tguanbo@jnu.edu.cn (B.-O.G.); 2 College of Physics & Optoelectronic Engineering, Jinan University, Guangzhou 510632, China; 3 College of Physics and Electronic Information & Henan Key Laboratory of Electromagnetic Transformation and Detection, Luoyang Normal University, Luoyang 471934, China; jiahong517@aliyun.com; 4 Longmen Laboratory of Luoyang, Luoyang 471934, China

**Keywords:** PbS quantum, temperature sensing, optical fiber

## Abstract

Glass ceramics (GCs) containing PbS quantum dots (QDs) are prepared for temperature sensing. Broadband emissions are detected in the GCs when PbS QDs are precipitated from the glasses, and emissions centers are modulated from 1250 nm to 1960 nm via heat treatments. The emission centers of GCs exhibit blue-shifts when environment temperatures increase from room temperature to 210 °C. Importantly, the shift values of emission centers increase linearly with the test temperature, which is beneficial for applications in temperature sensing. A temperature sensor based on PbS QDs GC is heat-treated at 500 °C for 10 h, possesses the highest sensitivity of 0.378 nm/°C, and exhibits excellent stability and repeatability at high temperatures (up to 210 °C). Moreover, GC fibers are fabricated by using the GCs as the fiber core. The sensitivity of the temperature-sensing sensor of the GC fibers is also demonstrated and the sensitivity is as high as 0.558 nm/°C. The designed PbS QDs GCs provide a significant materials base for the manufacturing of fluorescent temperature sensors and the GC fibers offer significant opportunities for temperature detection in complex, integrated and compact devices.

## 1. Introduction

Temperature sensors have gained growing interest because temperature is one of the most important physiological signals for reflecting the health status of life entities [1,2,3,4]. At present, thermistors, liquid-filled thermometers and thermos elements are usually used for temperature measurements [5]. However, the response rates of these commercial thermometers are slow due to the heat transfer needed to reach equilibrium, preventing applications in real-time thermometry. Optical temperature sensors have received widespread attention due to their intrinsic safety, high accuracy, fast response and high sensitivity [6]. For example, optical temperature sensors based on the difference between Stokes and anti-Stokes Raman signals make it possible to measure temperature with very high accuracy [7]. Fluorescent temperature sensors are easy to fabricate, can realize accurate and continuous temperature detection by measuring temperature-dependent fluorescence spectra, and are ideal candidates for real-time temperature sensing. Generally, the fluorescent properties of the sensors are deeply governed by host materials and luminescent centers. In past decades, a large number of luminescent materials, such as crystals, polymers, glasses and glass ceramics, have been synthesized to extensively study photothermal properties for potential application in real-time temperature sensing [8,9,10,11,12].

Glass ceramic (GC) is a significant composite containing a large number of glass phases as well as specific nanocrystals, which has been developed as a desirable luminescent material due to the outstanding advantage of glass featuring high optical transmittance and that of crystal exhibiting high-efficiency luminescence [13,14,15,16,17]. Moreover, GC maintains the unique fiber-drawing properties of glass, providing opportunities for the construction of efficient fiber sensors in compact and complex environments. In previous studies, a variety of rare-earth-ion-doped GCs have been used as luminescent materials for temperature detection [18,19,20,21]. However, temperature sensing is usually rooted in the intensity ratios of two upconversion emission peaks. Among them, Er^3+^–Yb^3+^-codoped GC is one commonly used luminescent material for temperature sensing due to the intense UC emissions related to the thermally coupled energy levels [22,23,24,25,26]. Actually, the intensity of the luminescence spectrum is usually inaccurate due to the response difference of the photoelectric detector. It is necessary to search for new GC materials for fluorescent temperature sensors.

In past decades, the photo-thermal properties of semiconductor QDs were widely investigated [27,28,29]. The red-shifts and blue-shifts of emission spectra were observed with an increase in test temperature. These indicated that semiconductor QDs are promising materials for temperature sensing. Owing to a narrow bandgap, large exciton Bohr radius and strong quantum limiting effect, PbS QDs exhibit tunable emissions in near- to middle-infrared regions [30]. More importantly, PbS QDs can be controllably precipitated in glass to prepare GCs. Previously, PbS QDs GCs have proved to be excellent candidates for temperature sensing because the bandgaps of PbS QDs were modulated via adjusting temperatures [31]. Furthermore, optical fibers are prepared by using PbS QDs GCs as core materials, providing significant opportunities for constructing novel fluorescent fiber temperature sensors. In past works, the photo-thermal properties of PbS QDs were also studied at low temperatures (from 0 K to room temperature) [32,33,34,35]. Here, the temperature-dependent luminescence properties of PbS GCs were investigated carefully at high temperatures (from room temperature to 210 °C), showing great potential for application in temperature sensing. Moreover, PbS QDs GC fibers were prepared and temperature-dependent properties were investigated, and they offer novel fiber temperature sensors for use in complex and compact environments.

## 2. Experimental

PbS GC samples were produced by a melt-quenching method followed by heat treatment processes. The molar composition of the precursor glass was 31SiO_2_-29B_2_O_3_-10ZnO-25Na_2_O-3BaO-1.0PbO-1.0ZnS. In a typical process, 30 g of the raw materials with the designed ratio was placed in an agate mortar and mixed thoroughly for 10 min. The mixed powder was put into a crucible and heated at 1100 °C in an electric furnace for 30 min. The precursor glass samples were fabricated by pouring the glass melt into an iron plate and rapidly casting into a slab. To prepare GC samples, the precursor glasses were heat-treated from 480 to 500 °C according to the thermal analysis results in our previous works [36]. These samples will be referred to as GC-480, GC-485, GC-490, GC-495 and GC-500 when the sample was heat treated at 480, 485, 490, 495 and 500 °C, respectively. The GC fibers were fabricated by using the PbS GCs as fiber cores via the melt-in-tube technique as reported in our previous works [37,38,39]. The samples were cut and polished as slabs with a thickness of 2 mm.

The amorphous state and crystalline phase of the GCs were identified by X-ray diffraction (XRD) on a D8 advance X-ray diffractometer (Bruker, Fällanden, Switzerland) with Cu/Kα (λ = 0.1541 nm) radiation. The XRD patterns of the samples were collected in the range of 10° < 2θ < 90° with a speed of 5°/min. The morphology and size distribution of the nanocrystals in GCs were measured by transmission electron microscopy (TEM) and high-resolution TEM (HR-TEM) (Tecnai G2, FEI, Hillsboro, OR, USA). Transmission spectra were measured by a UV/VIS/NIR spectrophotometer (Lambda-900, PerkinElmer, Waltham, MA, USA). The emission spectra of the samples were measured by using a spectrometer (OmniFluo533P-IR01, Zolix, Beijing, China) and using an 808 nm laser diode as the excitation source. In order to study the photo-thermal properties of PbS GCs and demonstrate their potential applications in temperature sensors, the samples were put in a temperature control box to measure emission spectra. The test temperatures were adjusted from room temperature to 210 °C.

## 3. Results and Discussion

**Figure 1:**
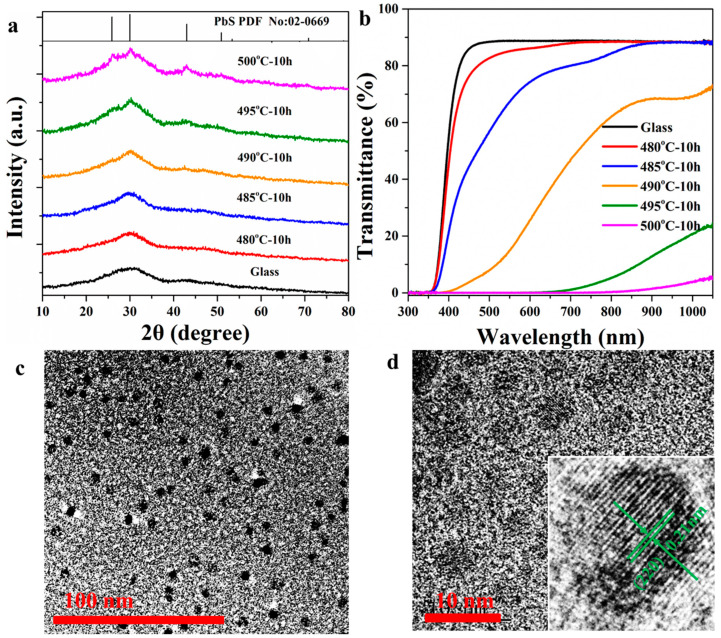
(**a**) XRD patterns of precursor glass, GCs and JCPDS Cards No: 02-0669 (PbS); (**b**) transmission spectra of glass and GCs; (**c**) TEM and (**d**) HR-TEM images of GC heat-treated at 500 °C for 10 h; the inset is an enlarged image of (**d**) containing only one crystal particle.

Figure 1a shows the XRD patterns of glass and GCs. Broad bands are observed, which are attributed to the amorphous phase of glass. Sharp peaks at 25.9°, 30.1°, 43.1° and 50.9° are observed in the XRD patterns of GCs, which match well with the diffraction of the (111), (200), (220) and (311) crystal facets of PbS (No: 02-0669) crystal, respectively. These results prove that PbS crystals have precipitated in the GC samples. The crystallization of PbS in the GC is governed by the small amount of PbO and ZnS. As a result, the crystallization ratio of PbS is small and the diffraction peaks are very weak in the XRD pattern of GCs. More crystals are precipitated in the GC when the heat treatment temperature increases to 500 °C, and the diffraction peaks in the XRD pattern are more obvious.

The transmission spectra of precursor glass and GCs are presented in Figure 1b. The precursor glass exhibits high transmittance (~80%) at a thickness of 2 mm. After heat treatments, the transmittance decreases dramatically due to optical scattering caused by PbS crystal particles. No absorption band is observed in the transmission spectrum of glass. However, intense absorption bands ranging from 700 to 1000 nm are observed in the transmission spectra of GCs. An 808 nm laser diode was used as the excitation source for the studies of optical properties of samples in our work according to the transmission spectra.

The TEM image of the GC heat-treated at 500 °C for 10 h is shown in Figure 1c. It is found that the nanocrystals are in situ precipitated among the glass matrix and the sizes of the crystal particles are below 10 nm. Crystal lattice fringes are observed in the HR-TEM image in Figure 1d. The interval of the crystal lattice fringes can be measured directly from the inset of Figure 1d and its value is about 0.21 nm, which corresponds to the (220) crystal facet of PbS crystal. The TEM and HR-TEM results indicate that PbS nanocrystals are precipitated from the glass matrix in the GC. PbS is an important semiconductor featuring excellent luminescent properties and it works as a quantum dot (QD) as the sizes are small [40].

**Figure 2:**
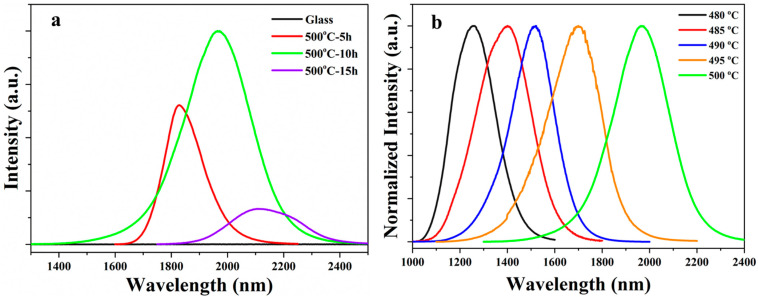
(**a**) Emission spectra of glass and GCs heat-treated at 500 °C for various hours; (**b**) compared emission spectra of GCs heat-treated at different temperatures for 10 h.

To study the luminescent properties of the as-made samples, emission spectra were measured by using a commercial 808 nm laser diode as the excitation source. No emission band is observed in the emission spectrum of glass as shown in Figure 2a. Broad emission bands are observed in the spectra of GCs heat-treated at 500 °C, which are attributed to the luminescence of PbS QDs, which is similar to a previous report [41]. The emission center is located at 1823, 1962 and 2115 nm when the samples were heated for 5, 10 and 15 h, respectively. Owing to the quantum confinement effect [42], the emission center of a QD is deeply governed by the sizes of crystal particles. Thus, the emission center exhibits a red-shift due to the enhancement in size of PbS crystal particles when the heat treatment time increases from 5 to 15 h. It is also found from Figure 2a that the emission intensity firstly increases because more PbS QDs are precipitated in the GC, reaching a maximum when the sample was heated for 10 h, and then decreases when the heat treatment time is further increased to 15 h due to the concentration quenching and self-absorption effect.

More importantly, the emission center of PbS QDs in the GCs can be modulated from 1250 to 1960 nm when the heat treatment temperature is adjusted from 480 to 500 °C as shown in Figure 2b. This result indicates that PbS QDs are precipitated in all GC samples and the crystal particles sizes increase when the heat treatment temperature is increased from 480 to 500 °C.

**Figure 3:**
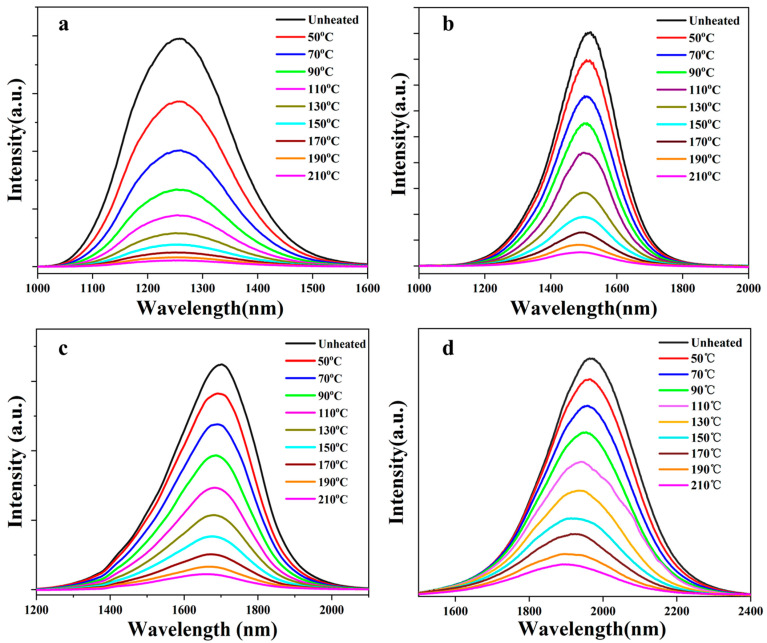
Temperature-dependent emission spectra of GCs heat-treated at (**a**) 480, (**b**) 490, (**c**) 495 and (**d**) 500 °C for 10 h.

The photo-thermal properties of GCs are studied by temperature-dependent emission spectra as shown in Figure 3. All of the emission intensities of the GCs monotonously decrease when the test temperature is increased from room temperature to 210 °C. This can be understood by considering the thermal quenching of luminescence. The phonon motions in the GCs are more intense when the temperature is increased, resulting in a higher probability of non-radiative transition and a decrease in emission intensity.

Moreover, the blue-shifts of spectra are observed in the spectra of GC-490, GC-495 and GC-500 (Figure 3b–d) as the test temperature increases from room temperature to 210 °C. These blue-shifts of emission spectra are ascribed to the electron–phonon coupling interaction of QDs [43]. For the PbS QDs, the bandgaps are enlarged due to the thermal expansion of the crystal lattices when the test temperature is increased [44], resulting in the blue-shifts of emission centers as shown in Figure 3b–d. However, almost no shift of spectra is observed in the spectra of GC-480.

**Figure 4:**
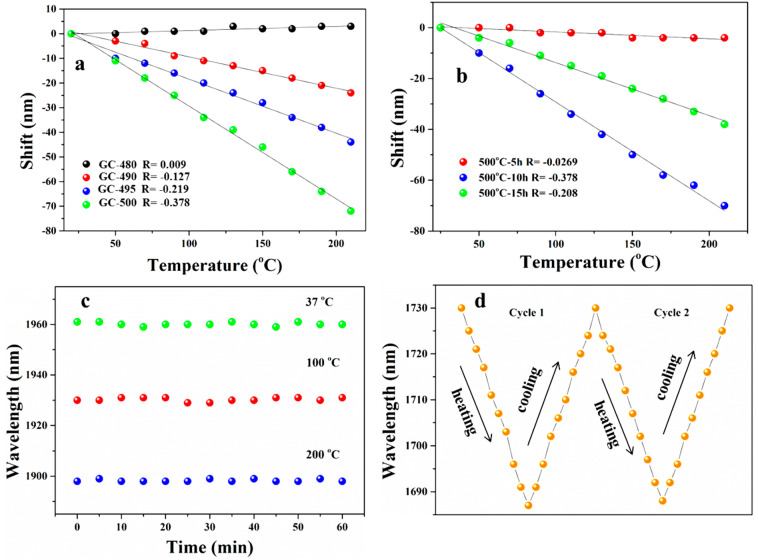
(**a**) Dependence of emission center shift of GCs heat-treated at various temperatures of test temperature. (**b**) Dependence of emission center shift of GCs heat-treated at 500 °C for different times of test temperature. (**c**) Dependence of emission center wavelengths of GC-500 on different heat-treat times at 37, 100 and 200 °C. (**d**) Emission center wavelengths of GC-495 recorded during two heating–cooling (25–210 °C) cycles.

Furthermore, Figure 4a plots the dependence of the shift values of the emission centers of the spectra dependent on the test temperature. The negative values indicate the shifts of the emission center to a shorter wavelength, that is, the blue-shifts of the emission spectra. It is found that the shift values of emission centers for GC-490, 495 and 500 all monotonously increase as the test temperature increases from room temperature to 210 °C. This is attributed to the increase in bandgaps of PbS QDs caused by the thermal expansion of the crystal lattices when the test temperature is increased [44]. It is also found that the shift values of GCs increase with the heat-treated temperatures of samples and the GC-500 sample possesses the largest shift values at all test temperatures. For the GC-480 sample, the sizes of QDs are small, the size-limited energy is intense and thermal expansion of the crystal lattices is weak [44]. No blue-shift is observed in the emission spectra. For GC heat-treated from 490 to 500 °C, the sizes and quantities of QDs are large and the thermal expansion of the crystal lattices is enhanced, but the size-limited energy is weaker, the bandgaps of PbS QDs are enlarged and the emission centers shift to short wavelengths, resulting in the blue-shifts of emission spectra.

Interestingly, the shift values of emission centers increase linearly with the test temperature, which is similar to previous reports [43], making GCs significant materials for application in temperature sensing. The slopes of the linearly fitted curves (R) of the shift values are plotted in Figure 4a. The R value increases from 0.127 to 0.378 when the heat-treated temperature increases from 490 to 500 °C. The shift values of GCs heat-treated at 500 °C for different times depend on the test temperatures and are presented in Figure 4b. The shift values increase when the heat-treatment time is increased from 5 to 10 h because more PbS QDs are precipitated in the GC. Then, the shift values decrease when the heated time was prolonged to 15 h due to dramatic self-absorption as larger QDs with various sizes are precipitated in the GC. Actually, the R value represents the sensitivity of temperature sensors [45]. The sensor based on the GC heat-treated at 500 °C for 10 h exhibits a high sensitivity of 0.378 nm/ °C, providing an excellent material for high-sensitivity temperature sensing.

To evaluate sensing properties, the stability and repeatability of the GC samples were studied, as shown in Figure 4c,d. The emission center measured at 37 °C (body temperature), 100 and 200 °C is located at 1960, 1932 and 1898 nm. When the GC sample was heated at these three temperatures, the recorded emission center wavelengths were almost consistent. The fluctuations of emission center wavelengths were ±1 nm. These results indicate that the sensor based on the designed GCs exhibits excellent stability and it is suitable for body-temperature detections and even high-temperature detections. Two cyclic temperature tests, each containing one heating and one cooling procedure, were conducted to evaluate the repeatability of the sensor based on the designed GC. The emission center shifts from 1730 to 1687 nm by heating the sample from 25 °C to 210 °C. Then, the emission center shifts from 1687 to 1730 nm by subsequent cooling of the sample from 210 °C to 25 °C. The center wavelengths at the same test temperatures are consistent with both heating and cooling processes. These indicate that the sensor exhibits excellent repeatability. Therefore, the designed GCs provide significant materials for temperature sensors, possessing excellent stability and repeatability.

**Figure 5:**
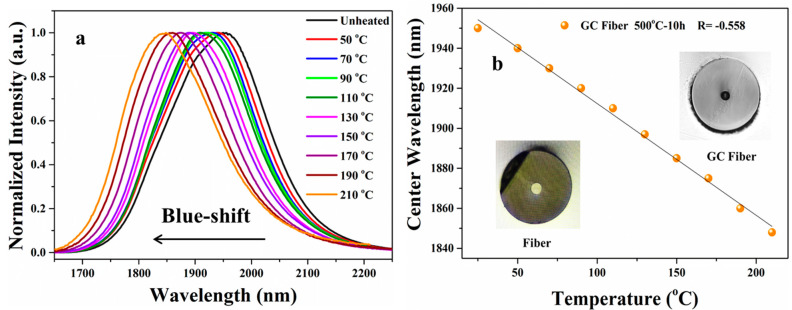
(**a**) Normalized emission spectra of a PbS GC fiber at various test temperatures. (**b**) Dependence of center wavelengths of the emission spectra of a GC fiber on test temperature. Insets, images of the fiber and the GC fiber.

Fiber-optic temperature sensors have received widespread attention due to their intrinsic safety, anti-electromagnetic interference, small size and remote detection. The temperature-dependent properties of fibers containing PbS QDs are investigated. The normalized emission spectra of a PbS GC fiber with a length of 5.0 cm and heat-treated at 500 °C for 10 h is presented in Figure 5a. Broadband emission is observed in the GC fiber, proving the precipitation of PbS QDs in the fiber. By increasing the test temperature, obvious blue-shifts of emission spectra are observed. The emission center shifts from 1951 nm to 1848 nm when the test temperature is increased from room temperature to 210 °C, as plotted in Figure 5b. The center wavelength decreases linearly with the test temperature. The slopes of the linear-fitted curves (R) is −0.558, indicating that the sensitivity of the sensor based on the GC fiber is 0.558 nm/°C. The GC fibers provide an excellent material for high-sensitivity fiber sensors, which can be used in complex and compact devices.

## 4. Conclusions

In conclusion, GCs containing PbS QDs were prepared by a melt-quenching method followed by heat treatments from 480 to 500 °C. Excited by an 808 nm laser, broadband emissions are observed in the GCs and the emissions centers are modulated from 1250 to 1960 nm by increasing heat treatment temperatures. It is found that the emission centers of GCs shifted to short wavelengths when the test temperatures increase from room temperature to 210 °C. The shift values of emission centers linearly increased with the test temperatures. A sensor was achieved based on GC-500 and the sensitivity was as high as 0.378 nm/°C. The temperature sensor possessed excellent stability and repeatability even when the environment temperature increased up to 210 °C. PbS QDs GC fibers were also prepared. The sensor based on the GC fiber exhibited a high sensitivity of 0.558 nm/°C. The designed PbS quantum dots GCs provide excellent photo-thermal materials and offer significant opportunities for the development of fiber temperature sensors for real-time temperature sensing in complex and compact environments.

## Data Availability

Data are contained within the article.
